# A consistent shift in VEGF determinations between two different ELISA batch numbers

**DOI:** 10.1038/sj.bjc.6601123

**Published:** 2003-07-15

**Authors:** K Werther, I J Christensen, H J Nielsen, the Danish RANX05 Corolectal Cancer Study Group

**Affiliations:** 1 Department of Surgical Gastroenterology 435, Hvidovre University Hospital, University of Copenhagen, Denmark; 2 The Finsen Laboratory, Rigshopitalet, 49 Strandboulevarden, DK-2100 Copenhagen, Denmark

**Sir**,

In February 2002, we published an article in the *Br J Cancer* concerning the prognostic impact of matched preoperative plasma and serum VEGF in patients with primary colorectal carcinoma (Werther *et al*, 2002).

In this publication, serum VEGF concentrations were determined in 524 patients with colorectal cancer (CRC), and in 50 healthy blood donors (HRD). All serum VEGF concentrations were determined with the commercially available human VEGF ELSIA kit (R&D Systems, Minneapolis, MN USA, Cat No: DVE00), according to the instructions given by the manufacturer. However, different batch numbers of the ELISA kit were used in the two groups. In the CRC patient group, serum VEGF concentration were determined with ELISA kits with ‘old’ batch numbers, while VEGF determination in HBD were performed with ELISA kits ‘new’ batch numbers.

In order to evaluate preoperative serum VEGF as a prognostic maker, serum VEGF concentration were scored as low if VEGF was less than or equal to the 95th percentile of normal controls (533 pg ml^−1^) or otherwise scored as high. In addition, the patients were grouped into three strata by the 10th and 90th or the 25th and 75th percentiles of their serum VEGF concentrations.

Recently, we discovered a significant increase in VEGF levels when ELISA kits with the ‘new’ batch number were used. In order to investigate this shift, a number of preoperative serum samples were randomly selected and were reanalysed with the ELISA kits with the ‘new’ batch number. This re-evaluation showed that the VEGF levels in the preoperative serum samples were significantly higher when determined with a kit with the ‘new’ batch number. In addition, it was shown that there was a strong correlation between the VEGF concentrations obtained from the two different batches. These results demonstrate a consistent and systematic difference between the two batches.

Therefore, we find it necessary to inform that the serum VEGF cutoff levels used in the BJC publication may not be accurate.

In an earlier study, we have determined VEGF in 91 healthy controls with the ‘old’ batch ELISA's (Werther *et al*, 2000). In this study, the 95th percentile of normal controls was 465 pg ml^−1^, compared to 533 pg ml^−1^ in the *Br J Cancer* publication. We believe that it may be more accurate to use this cutoff level.

Therefore, we have applied the new cutoff levels to the VEGF determination in the 524 CRC patients. Fortunately, the application of this new cutoff level (465 pg ml^−1^) does not change the conclusions in the article published in BJC.

In the *Br J Cancer* article, we also investigated the prognostic impact of preoperative plasma VEGF concentrations. However, all plasma VEGF determinations (both CRC patients and HBD) were performed with ELISA kits with the ‘new’ batch number. Therefore, in the plasma VEGF calculations and figures, the cutoff level we used should be accurate.

We feel it is important to inform *Br J Cancer* and its readers about this finding.
